# Assessment of prevalence and motivators for family planning utilisation among youth students in higher learning institutions in Dodoma, Tanzania: A cross sectional study

**DOI:** 10.1371/journal.pone.0308939

**Published:** 2024-11-27

**Authors:** Upendo Munuo, Fabiola Vincent Moshi

**Affiliations:** 1 Department of Clinical Nursing, School of Nursing and Public Health, The University of Dodoma, Dodoma Tanzania; 2 Department of Nursing Management and Education, School of Nursing and Public Health, The University of Dodoma, Dodoma Tanzania; BHU: Bule Hora University, Institute of Health, ETHIOPIA

## Abstract

**Background:**

Sub-Sahara Africa (SSA) has the greatest unmet need for family planning use, but young people are reported to under-utilise family planning services compared to other groups of women of the reproductive age in the region. Thus, promoting contraceptive services use among adolescents is vital in achieving universal access to sexual and reproductive health services.

**Objective:**

The main objective of this study was to assess the prevalence and motivators for family planning services utilization among youth students in higher learning institutions in Dodoma, Tanzania.

**Methods:**

An analytical cross-sectional study was conducted among 421 randomly selected youths in higher learning institutions in Dodoma. A self-administered structured questionnaire adopted from previous studies was used to collect data. Bivariate and multivariate logistic regression analysis using SPSS version 25 established association between variables. Statistical significance was declared at 95% confidence interval with α<0.05 and strength of association was reported by Odds Ratio (OR) and Adjusted Odds Ratio (AOR).

**Results:**

Majority of the participants 221 (52.5%) were females, and most of them 320 (76.0%) were aged between 21 to 24 years. Of all participants, bachelor’s degree scholar were 257 (61.0%). About 305, (72.4%) participants reported to have had sexual intercourse in the past 12 month. Of these 50.5% had their first intercourse at the age between 15 to 19 years. Most participants 239 (56.8%) reported to have used contraceptive method in the past 12 months. After adjusting for confounders (participants’ socio-demographic characteristics), the motivators for family planning use among youth students were: living with relatives (AOR = 2.904, p 0.006), adequate FP knowledge (AOR = 2.230 p 0.003), positive perception (AOR = 1.772, p 0.025) and discussing FP matters with sexual partners (AOR = 6.045, p <0. 001).

**Conclusion:**

This study highlights a significant unmet need for family planning services among youth students in higher learning institutions in Dodoma, Tanzania, reflecting broader trends in Sub-Saharan Africa. Despite high sexual activity, family planning utilization remains suboptimal. Key motivators for using family planning services include living with relatives, having adequate knowledge, positive perceptions, and discussing family planning with sexual partners. These findings emphasize the need for targeted interventions focusing on education, fostering positive perceptions, and encouraging open partner discussions to enhance family planning utilization among adolescents, contributing to universal access to sexual and reproductive health services.

## Introduction

World Health Organization (WHO) delineates youth as the stage when a person is no longer a child but is not yet an adult. Age wise, adolescence is considered from 10 to 24 years [1]. Worldwide, 1.2 billion out of the estimated 3 billion young population is aged less than 25 years [2]. In sub-Sahara Africa, approximately 1 in every 3 individuals is aged between 10 to 24 years [2]. Young girls are the most vulnerable group for not using Sexual Reproductive Services, including family planning, yet they are given diminutive attention, especially in sub-Saharan Africa. This is due to the fact that they are victims of early marriage, teenage pregnancy, unwanted and unintended pregnancy, and sexual transmitted infections concomitant with early unveiling to unsafe sexual behaviours [3].

In low and middle income countries such as Tanzania, one-third of the women were married at the age under 18 years, whereas 1 in 9 are wedded at 15 years old [3]. It is projected that 11% of births befall among youths globally whereby more than 90% of these births happen in low and middle income countries [3]. Sub-Saharan Africa is amongst the regions with uppermost teenage pregnancy rates and yet with the lowest rates of family planning (FP) utilisation [4].

In Tanzania, particularly, adolescents comprise almost one quarter (24%, or 12.8 million) of the total population [5]. The matter of health services provision such as reproductive health, nutrition services and screening services to youth is a concern, which needs attention from the government and different stakeholders. Use of sexual and reproductive health services (SRHs) including FP services among youths is an essential strategy in preventing teenage pregnancies, unsolicited and unintended pregnancy, unprotected sexual intercourse which can result to reduction of maternal morbidity and mortality and STIs risks [4].

Promoting FP services utilisation among adolescents is crucial in attaining universal access to sexual and reproductive health services [6]. Sub-Sahara Africa (SSA) has the paramount unmet need for family planning utilisation [7], and young people are reported to under-utilise FP services compared to other groups of women of the reproductive age in the region [4].

According to WHO guideline entitled *“Making Health Services Adolescent Friendly”*, SRH/FP services targeted for adolescents should abide with the quality elements which comprise: accessibility, acceptability, equitable, appropriate, and effective [1]. Adolescents need to be offered FP services in the environment which favours their privileges and they should be made to feel welcomed by health care providers [8].

In Tanzania, predominantly in Dodoma, the existing and recommended FP methods for youth and young girls are oral contraceptives pills such as Combined oral contraceptives (Microgynon), Progestin-only pills (Microval), Emergency contraceptives, Injectables such as Depo medroxyprogesterone acetate (DMPA), Implants that include Double-rod implant (Jadelle) and Single-rod implant (Implanon), Intra uterine devices (Copper T 380A) [9]. Additionally, there are natural methods such as Standard Days Methods (SDM), Lactational Amenorrhea Method (LAM) and Cycle Beads. Also, barrier methods such as male condoms and female condoms offer both protection against unwanted pregnancy and sexually transmitted infection including HIV/AIDS (dual protection). However, those available FP methods if consistently and properly utilised, will avert unplanned or unwanted pregnancies and thus curtailing avertable maternal morbidity and mortality among youth girls [9]. It is anticipated that universal access and use of family planning methods among adolescents can results to reduction of 2.1 million unplanned and or unwanted births, 3.2 million unsafe abortions, and 5600 maternal demises each year [10]. Use of family planning methods additionally provides adolescents with a chance to make informed decisions about when they want to have children [9].

Despite these benefits, the youth face numerous issues which hinder their use of FP services. Young people have reported to receive FP services with lower satisfaction to the service they received from healthcare providers elsewhere in SSA [4]. According to 2015/16 TDHS, many young people were reported to have a limited education concerning SRH services, including FP methods. As a consequence, they continued to engage in unsafe sexual practices and behaviours such as multiple and concurrent partnerships and inconsistent condom use [11].

Teenage pregnancy remains a challenge to young people in schools, colleges and the community at large. This may be contributed to by either insufficient use of FP services which is considered a vital strategy towards reducing preventable teen pregnancies, and other associated complications [4, 8].

The youths from higher learning institutions tend to engage in unplanned and unprotected sexual activities easily, which is thought to be attributed by lack of strict parental environment [12]. This behaviour may subject the youth to unintended pregnancies, abortions and sexual transmitted infections [12–15]. Similarly, unintended pregnancies may bring in social consequences to the students, such as school dropout, economic hardships, social abandonment and neglect [16]. Moreover, evidence from the study conducted in universities from Dar es Salaam Region revealed that unwanted pregnancy rate was 27% and abortion rate was 54.6% [12].

However, it is not well known what might be the motivators for university youth students to use FP services in Tanzania, particularly in Dodoma region. Similarly, majority of the studies studied female undergraduates, while others confined their studies to a single institution, thus leaving some questions unanswered [13, 14]. Therefore, the current study aimed to assess the prevalence and motivators for the utilisation of family planning services among the youths in higher learning institutions in Dodoma Region.

## Materials and methods

### Study area

The study was undertaken in Dodoma City, which has a size of 2769 square kilometres with 453,844 households, 41 wards 170 streets 42 villages and 89 hamlets. The city is situated at the geographical centre of the country on the vital Central Railway line, and main crossroad of the National East West trunk road and the famous north to south Cape to Cairo Great North Road, which passes in Tanzania through Mbeya, Iringa, Dodoma, Babati and Arusha. The city is about 465 kilometres from Dar-es-Salaam, and 425 kilometres from Arusha, located at 6^oo^ south of the Equator. The city has a total population of more than 2.6 million of people with growth rate of 3.1% [17–19] and total fertility rate of 5.7% [19]. Furthermore, there are eight (8) higher learning institutions in Dodoma city, namely: The University of Dodoma (UDOM), St John’s University of Tanzania (SJUT), College of Business Education (CBE), and Institute of Rural Development Planning (IRDP). Others are Local Government Training Institution Hombolo—Dodoma, Capital Teachers College, Dodoma Institute of Health and Allied Sciences (DIHAS), and DECCA College of Health and Allied Sciences. Most of these learning institutions offer RMNCAH services, including free family planning that includes UDOM, SJUT, DIHAS and DECCA. Four higher learning institutions that were selected for the study were: UDOM, SJUT, CBE and IRDP. These were selected randomly.

### Study design and population

The study design was analytical cross-sectional study with quantitative approach. It was analytical because the regression model was used to control for confounding variables between the independent and dependent variables. All possible independent variables were entered into the regression model and all independent variables, which showed a significant relationship, were taken as the motivators of family planning utilisation. Unmarried youth students from the selected higher learning institutions aged between 18–24 years were included in the study. On the other hand, the study excluded married youth students of the same age range (18–24 years) and those who were unwilling to participate in the study.

### Sample size calculation and sampling techniques

#### Sample size calculation

The Cochran’s (1977) [20], formula was used to determine the minimum sample size required for the study. According to this formula, n = desired sample size, z = critical value at 95% confidence level corresponding to 1.96, d = marginal error (desired level of precision which is 5%), and p = prevalence of FP use among youth students (47%), [14].


$$n=\frac{z^{2}P(100-P)}{d^{2}}=\frac{1.96^{2}\times 47(100-47)}{5^{2}}=383$$

To cater for potential unresponsiveness, 10% of the minimum sample size was added up as shown below:

- 10/100*383 = 38.3 ≈ 38 + 383 = 421.

Therefore, the total sample size was 421 participants.

#### Sampling techniques

The simple random techniques were used in the selection of 4 higher learning institutions and 421 youth students. The proportional allocation formula was also used in determining the required sample per institution. This was worked out following this approach: (i) determined the number of the eligible youth students from each institution; (ii) employed the formula below to calculate representative sample size from each of the selected institution; (iii) a simple random sampling technique was used to obtain the participants from each of the selected institutions [21].


$$n_{i}=\left(\frac{n}{N}\right)N_{i}$$

Where: n = total sample size to be selected, N = total population, N_i_ = total population of each facility, and n_i_ = sample size from each facility (Table 1).

**Table 1:** Participants distribution per institution.

Institution name	Population per institution (N_i_)	Sample size per institution (n_i_)	Percent (%)
St. John’s University of Tanzania	3900	63	15.0
UDOM (College of Education)	8200	133	31.6
College of Business Education (CBE)	3300	53	12.6
Institute of Rural Development Planning (IRDP)	10,653	172	40.8
**Total (N)**	**26,053**	**421 (n)**	**100**

#### Data collection tool and procedure

Self-administered questionnaire adopted from the previous study [20] was used in collecting data from the research participants. The questionnaire comprised of six sections: (i) Demographic characteristics, (ii) Individual sexual characteristics, (iii) Knowledge on FP (iv) Perception on FP use, (v) FP services utilisation, and (vi) Health service provision environment. The questionnaire was constructed in English, comprising both open and closed questions. Data were collected for a period of one month from 11^th^ April to 6^th^ May 2022 whereby the questionnaires were distributed to participants to fill in a separate room for confidentiality under the supervision of the assistant researcher.

#### Validity and reliability of data collection tool

The validity of the current study was based on content validity. It was also based on a review of the previous study to create a pertinent questionnaire. Two research assistants were trained on the data collection tool for two days. The individuals recruited as research assistants were those with prior experience in providing FP services. Next, 10% of the actual sample size from the Dodoma Institute of Health and Allied Sciences (DIHAS) was used in a pilot study. This institution was not a part of the actual study participants. The questions that appeared to have unclear meaning were corrected. Following that, FP specialists were consulted to assess the questionnaire’s coherence and applicability. The instrument was then adjusted in order to measure the necessary parameters.

Additionally, A Cronbach’s alpha test was conducted for the knowledge and perception variables in order to determine the internal consistency of the data collection instruments. The results fell within an acceptable reliability range, with 0.745 and 0.825, respectively. Furthermore, the principal investigator remained accessible throughout to oversee the data collection procedure and provided the necessary assistance to the research assistants.

#### Data processing and analysis

Collected data were cleaned and analysed using SPSS version 25.0 software package. At CI of 95% both descriptive and binary logistic regression were performed. Binary logistic regression was used since the dependant variable was dichotomous. After running statistical relationship, the variables with p-values less than or equal 0.2 were taken for further analysis, whereby the Percentages, P-values, Odds Ratio (OR) and Adjusted Odds Ratio (AOR) were presented respectively.

#### Ethical approval and consent to the participants

Ethical clearance was obtained from the University of Dodoma Research Committee with Ref. No. MA.84/261/02/’A’/91. Likewise, permission to conduct research was sought from the Regional Administrative Secretary (RAS) of Dodoma region with Ref. no. DA.122/467.01C/134. In addition, the principal investigator presented the authorisation letter to the respective higher learning institutions where the study was to be conducted.

Participants were provided with informed consent form prior to the conducting of the study whereby, an individual written informed consent was obtained from the study participants after being fully informed about the aim of the study and the processes involved. Participants were guaranteed about privacy and confidentiality.

## Results

### Socio-demographic characteristics of the participants

Table 2 shows that 421 participants were involved in this study with a response rate of 100%. The participants’ mean age was 21.67 (±SD = 1.58) years with 18 years minimum age and 24 years being a maximum age while the prominent age group of 320 participants (76.0%) ranged between 21 and 24 years. Majority of the participants (221, which equals to 52.5%) were females. In addition, 257 (61.0%) participants were studying bachelor’s degree of which 177 (42.0%) were first year students. Many participants (358, equal to 85.0%) were pursuing non-health related programmes. Moreover, more than half of the participants (249) were living in off campuses. With regard to marital status, most of the participants (91.4%) were single. In addition, 287 (68.2%) participants were living with both parents. This study noted that 251 (59.6%) participants discussed family planning issues in their families.

**Table 2:** Socio demographic characteristics of study participants (n = 421).

Characteristics	Frequency (n)	Percent (%)
Sex		
Female	221	52.5
Male	200	47.5
Age (in years)		
18–20	101	24
21–24	320	76
Education Level		
Certificate	76	18.1
Diploma	88	20.9
Undergraduate	257	61.0
Year of study		
First year	177	42.0
Second year	98	23.3
Third year	146	34.7
Programme		
Health	63	15.0
Non- health	358	85.0
Residence		
In campus	172	40.9
Off campus	249	59.1
Marital status		
Single	385	91.4
Cohabiting	36	8.6
Living with who at home		
Both parents	287	68.2
Single parent	76	18.0
Other relatives	58	13.8
Discuss FP issues with the family member		
Yes	251	59.6
No	170	40.4

### Sexual characteristics of the study participants

Table 3 shows sexual characteristics of youth students. The results show that 307 (72.9%) participants reported to have had sexual intercourse before the conduct of this study. Among those who have had sexual intercourse, majority (196, 63.8%) reported to have sexual intercourse with one person in the past 12 months, while 111 (36.2%) participants had sexual intercourse with more than one person. Similarly, it was noted that those who reported to ever had sexual intercourse, 155 (50.5%) had their first sexual intercourse at the age of 15–19 years while 16 (5.2%) participants had it at the age of <15years. It was also found that over half of the participants, 212 (50.4%) discussed FP methods with their sexual partners while less than half of the participants (n = 147, 34.9%) had discussed FP services with health workers. Moreover, 247 (58.7%) participants had exposed to mass media to access information about FP services and the main source of information was television (reported by 182, which is equal to 43.2%). Moreover, majority of the participants (n = 287, 68.2%) reported to have been influenced by their friends to utilise FP services.

**Table 3:** Sexual characteristics of the study participants (n = 421).

Characteristics	Frequency (n)	Percent (%)
Number of sexual partner (s)		
None	51	12.1
One	251	59.6
More than one	119	28.3
Ever had sexual intercourse		
Yes	307	72.9
No	114	27.1
No. of persons had sexual intercourse with for those who had ever have sexual intercourse		
One	196	63.8
More than one	111	36.2
Age at first sexual intercourse		
< 15 years	16	5.2
15–19	155	50.5
20–24	136	44.3
Discuss FP methods with your sexual partner		
Yes	212	50.4
No	209	49.6
Discuss FP methods with health workers		
Yes	147	34.9
No	274	65.1
Exposed to mass media to access information on FP Service		
Yes	247	58.7
No	174	41.3
Source of information		
Radio	163	38.7
Television	182	43.2
Others	33	3.6
Influence utilisation of FP services		
Parents decision	170	40.4
Partner	231	54.9
Religious beliefs	70	16.6
Influence from friends	287	68.2
Cultural beliefs	22	5.2
Cost of the FP services	24	5.7

### Prevalence of family planning services utilisation among higher learning youth students

**Fig 1:**
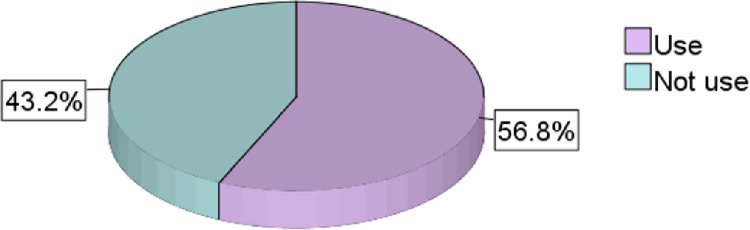
FP use among youth students from HLIs in the last 12 months.

In this study, the participants were assessed on their utilisation of FP services. According to the research results, majority of the participants 239 (56.8%, 95% CI = 51.9, 61.6) reported to have ever used any method of FP methods in the past 12 months (Fig 1).

### Different family planning methods used by the study participants

**Fig 2:**
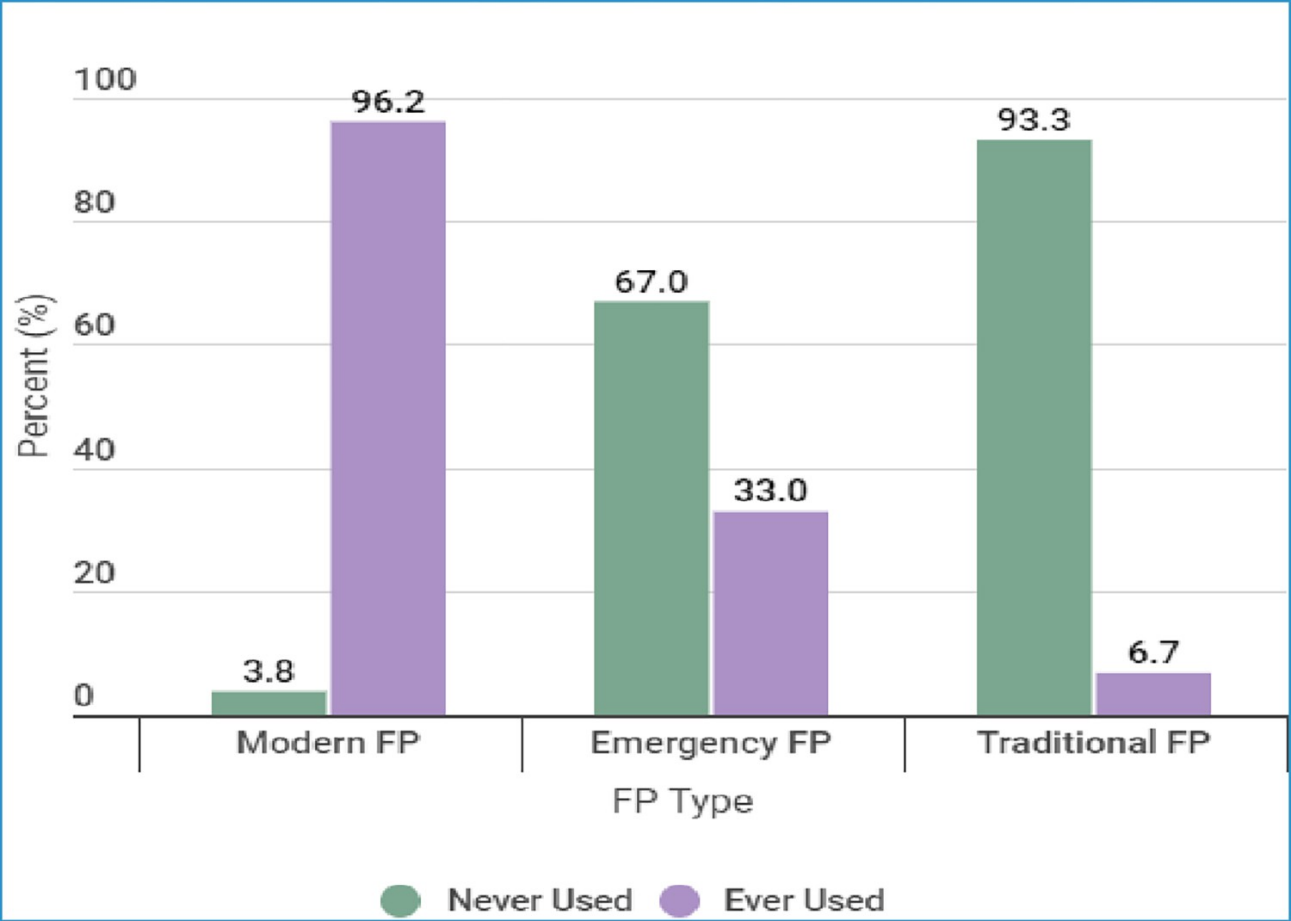
FP use by type among youth students from HLIs.

Out of the 239 participants who used FP methods, 230 (96.2%) were using modern FP, 79 (33.0%) were using emergency contraceptives while 16 (6.7%) were using traditional FP methods in the past 12 months. This information is summarised in Fig 2.

**Fig 3:**
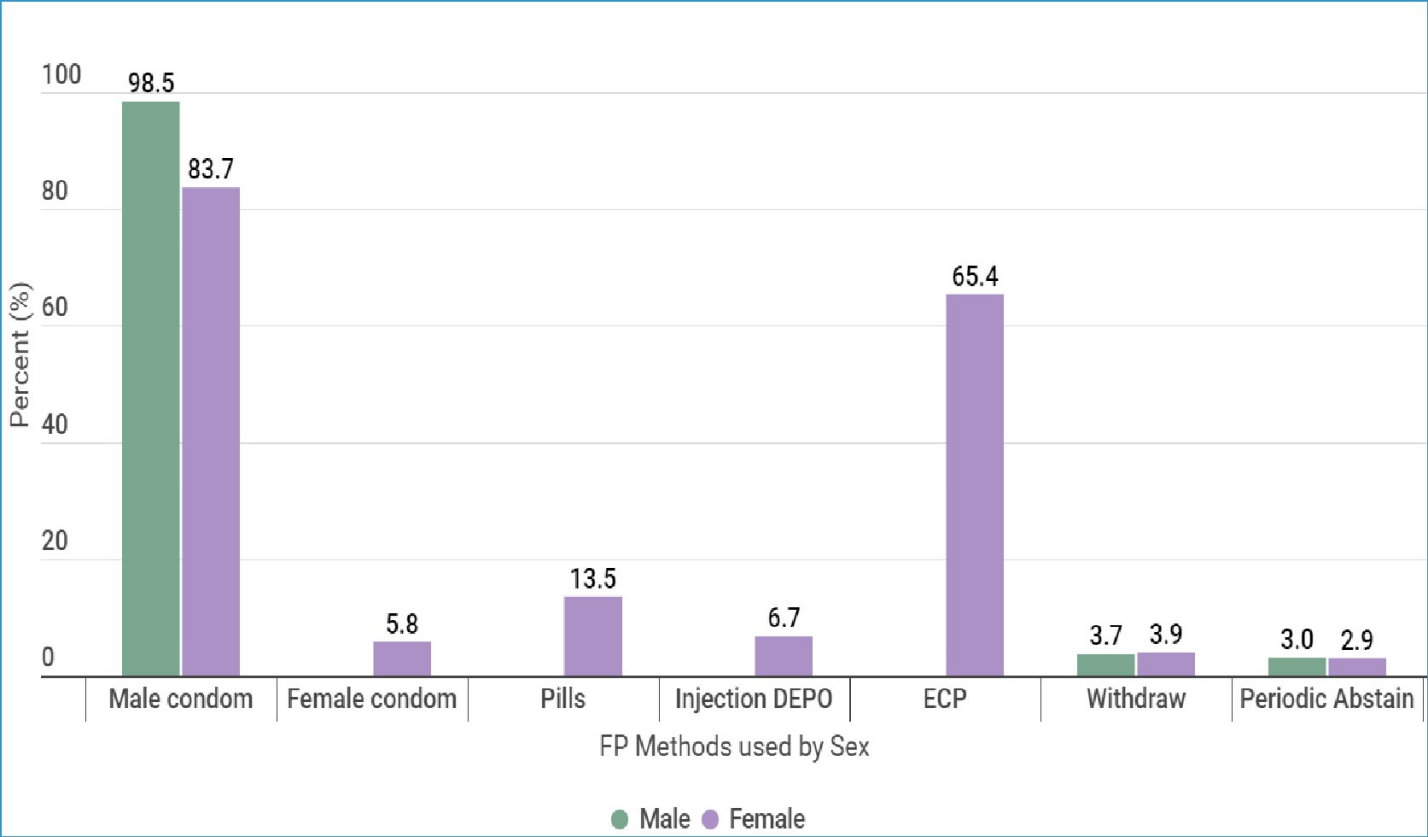
FP method used by sex among youth students from higher learning institutions.

With regard to FP methods used, majority of users, 133 (98.5%) males and 87 (83.7%) females were using male condoms. Emergency contraceptive was the second highest method used by female users, accounting for 65.4%. The least used FP method was periodic abstinence, which was reported by 4 (3%) male 3 (2.9%) female participants. The findings regarding other methods are as presented in Fig 3.

### Motivators of family planning services utilisation among higher learning youth students

To determine the motivators of family planning services utilisation, initially; chi square tests were performed in cross-tabulation to establish the relationship between FP services utilisation against socio-demographic characteristics, FP knowledge, FP perception and FP provision environment.

### Relationship between the participants’ socio-demographic characteristics and family planning services utilisation

The results from Chi-square test revealed that sex, age, marital status, persons living with the respondents at home, discussion of FP with the partner, FP knowledge and perception on FP use; were statistically and significantly associated with FP utilisation. The results reveal that, male students were found to have utilised FP services more (67.5%) than female students (47.1%), (χ2 = 17.875, p <0.001). Students aged between 21 to 24 years were found to have utilised FP services by higher per cent (60.9%) than those ranging between 18 to 20 years (43.6%), (χ2 = 9.441, p 0.002). In addition, participants who stayed off campus had utilised FP services more (59.4%) compared to those who were staying in campus (52.9%), (χ2 = 1.768, p 0.184). Moreover, as Table 4 displays, it was noted that the utilisation of FP services was higher among cohabiting students (83.3%) than those who were single (54.3%), (χ2 = 11.319, p 0.001).

**Table 4:** Relationship between the participants’ socio-demographic characteristics and family planning services utilisation (n = 421).

Characteristics	FP services utilisation		χ2	p-value
	Used n (%)	Not used n (%)		
Sex				
Male	135 (66.5)	65 (32.5)	17.875	<0.001
Female	104 (47.1)	117 (52.9)		
Age (in years)				
18–20	44 (43.6)	57 (56.4)	9.441	0.002
21–24	195 (60.9)	125 (39.1)		
Programme				
Health related	39 (61.9)	24 (38.1)	0.796	0.372
Non- health related	200 (55.9)	158 (44.1)		
Education Level				
Certificate	40 (52.6)	36 (47.4)	3.906	0.142
Diploma	58 (65.9)	30 (34.1)		
Undergraduate	141 (54.9)	119 (45.1)		
Residence				
In campus	91(52.9)	81 (47.1)	1.768	0.184
Off campus	148 (59.4)	101 (40.6)		
Year of study				
First year	96(54.2)	81(45.8)		
Second year	54(55.1)	44(44.9)	1.618	0.445
Third year	89(61.0)	57(39.0)		
Marital status				
Single	209 (54.3)	176 (45.7)	11.319	0.001
Cohabiting	30 (83.3)	6 (16.7)		
Discuss FP issues with the family members				
Yes	151 (60.2)	100 (39.8)	2.910	0.088
No	88 (51.8)	82 (48.2)		
Persons you are living with at home				
Both parents	153 (53.3)	134 (46.7)	7.223	0.027
Single parent	44 (57.9)	32 (42.1)		
Other relatives	42 (72.4)	16 (27.6)		
Discuss FP methods with your sexual partner				
Yes	169 (79.7)	43 (20.3)	91.629	<0.001
No	70 (33.5)	139 (66.5)		
FP knowledge				
Inadequate	47 (38.2)	76 (61.8)	24.386	<0.001
Adequate	192 (64.4)	106(35.6)		
FP use perception				
Positive	176 (63.3)	102 (36.7)	14.263	<0.001
Negative	63 (44.1)	80 (55.9)		

Similarly, it was found that FP services utilisation was higher among students who were living with relatives other than their parents (72.4%) compared to those who were living with both parents (53.3%) and those who were living with single parents (57.9%), (χ2 = 7.223, p 0.027). On the other hand, it was noted that participants who discussed FP matters with their sexual partners were more users of FP services (79.7%) than those who did not (33.5%), (χ2 = 91.629, p <0.001). Furthermore, participants with adequate FP knowledge were utilising FP services more (64.4%) than those with inadequate FP knowledge (38.2%), (χ2 = 24.386, p <0.001). Moreover, it was found that FP services utilisation was higher among participants with positive perception (63.3%) than those who had negative perceptions (44.1%), (χ2 = 14.263, p <0.001). This information is captured in Table 4.

### Relationship between health services provision environment and FP services utilisation

The results from a chi-square test show that discussing FP matters with HCPs, adequate time for discussion and distance from residence to nearest health facility were statistically and significantly associated with FP utilisation among youth students. Table 5 presents more details regarding this aspect.

**Table 5:** Relationship between the health services provision environment and FP utilisation (n = 421).

Variable	FP services		χ2	p-value
	Used n (%)	Not used n (%)		
Discuss FP methods with health workers				
Yes	106 (72.1)	41 (27.9)	21.655	<0.001
No	133 (48.5)	141 (51.5)		
Healthcare providers allow adequate time for discussion with youth				
Yes	184 (61.7)	114 (38.3)	10.288	<0.001
No	55 (44.7)	68 (55.3)		
Distance from the place of residence to nearest health facility				
Within 5 km	159 (60.2)	105 (39.8)	7.636	0.022
5–10 km	59 (56.7)	45 (43.3)		
>10 km	21 (39.6)	32 (60.4)		

Participants who had discussed FP matters with HCPs were more likely to utilise FP services (72.1%) than those who did not discuss FP matters with HCPs (48.5%), (χ2 = 21.655, p <0.001). Moreover, participants who were given adequate time to discuss FP issues with HCPs and their peers were found to utilise FP services more (61.7%) than their counterparts (χ2 = 10.288, p <0.001). Furthermore, it was noted that participants who were living near health facility (within 5kms) had more chances to utilise FP services (60.2%) than those who were living far (more than 5kms) from the health facility (χ2 = 7.636, p 0.022).

### Motivators for family planning services use among the higher education youth students

The current study has revealed that participants who were living with relatives other than their parents were more likely to utilise FP services (AOR = 2.904) compared to those who were living with their parents (95% CI = 1.367, 6.169, p 0.006). Likewise, participants with adequate FP knowledge had higher chance of utilising e FP services (AOR = 2.230) than those with inadequate FP knowledge (95% CI = 1.316, 3.777, p 0.003). It was noted further that having a positive perception towards FP use increases the chance for FP services utilisation (AOR = 1.772) compared to those with negative perception (95% CI = 1.076, 2.918, p 0.025). Similarly, participants who were discussing FP matters with their sexual partners were more likely to utilise FP services (AOR = 6.045) compared to those who were not doing so (95% CI = 3.600, 10.150, p <0.001). Table 6 presents the details of these findings.

**Table 6:** Logistic regression analysis for determining the motivators for FP services use among the participants (n = 421).

Characteristics	OR (95% CI)	P-value	AOR (95% CI)	P-value
Sex				
Female	Ref	-	-	-
Male	2.337 (1.571,3.474)	<0.001	1.988 (1.223,3.233)	0.006
Age (in years)	1.299 (1.144,1.475)	<0.001	1.033 (0.880,1.213)	0.690
Marital status				
Single	Ref	-	-	-
Cohabiting	4.211(1.713,10.348)	0.002	2.417 (0.879,6.641)	0.087
Persons you are living with at home				
Both parents	Ref	-	-	-
Single parent	1.204 (0.722,2.007)	0.476	1.466 (0.786,2.737)	0.229
Other relatives	2.299 (1.236,4.277)	0.009	2.904 (1.367,6.169)	0.006
FP knowledge				
Inadequate	Ref	-	-	-
Adequate	2.929 (1.897,4.521)	<0.001	2.230 (1.316,3.777)	0.003
FP use perception				
-ve perception	Ref	-	-	-
+ve perception	2.191(1.453,3.303)	<0.001	1.772 (1.076,2.918)	0.025
Discuss FP methods with sexual partner				
No	Ref	-	-	-
Yes	7.804(5.021,12.131)	<0.001	6.045 (3.600,10.150)	<0.001
Discuss FP methods with health workers				
No	Ref	-	-	-
Yes	2.741(1.781,4.219)	<0.001	1.293 (0.739,2.264)	0.368
HCPs allow adequate time for discussion with youth				
No	Ref	-	-	-
Yes	1.996(1.304,3.053)	0.001	1.329(0.786,2.247)	0.288
Distance from the place residence to nearest health facility				
>10 kms	Ref	-	-	-
5–10 kms	1.998(1.019,3.918)	0.044	1.349 (0.593,3.066)	0.475
Within 5 kms	2.307(1.262,4.218)	0.007	1.364 (0.649,2.867)	0.413

Key: OR = Odds Ratio, AOR = Adjusted Odds Ration, CI = Confidence Interval

## Discussion

The current study found that over half of the participants had used contraceptives in the past 12 months before the conduct of this study. In addition, the factors for FP use were contraceptive knowledge, positive perception and conducive environment. The findings of the current study reveal that the FP utilisation was quite higher compared with other studies which documented a lower utilisation in Uganda and Kenya [22–24]. The higher prevalence might be due to contraceptive knowledge, positive perception and conducive environment. The similarities between the studies are due to the fact that both studies were conducted in the similar setting and the study participants had the same characteristics.

Surprisingly, the discrepancy between sexual activity and family planning (FP) service utilization among study participants highlights a significant gap in reproductive health care. Despite 73% of participants being sexually active, only 56% reported using FP services. This disparity underscores several challenges and barriers to accessing and adopting FP methods effectively. The unmet need in this population can be explained in number of reasons, one of which may be lack of motivation to use FP among youth students. Studies have reported that making the FP services user friendly and easily accessible can motivate youth to use the services [25].

In addition, this study found that male condoms were the most used FP method by the majority of the youth students. Similar findings have been reported by other studies that were conducted in Lesotho, Uganda and Kenya [22, 23, 26]. However, this finding contrasts with other studies that have identified oral contraceptives, withdrawal methods, and emergency contraceptives as the most commonly known and utilized family planning methods [13, 27].

Contrary to these findings, a poor practice towards FP was reported among tertiary students in Namibia, where three-quarters of the students showed to have inadequate uptake of contraceptive methods [28]. It was reported that the youth tended to fear stigmatisation from the healthcare providers who often discouraged them from seeking services [22].

Furthermore, the study reveals that more than half of the female family planning users reported having used emergency contraceptives in the past 12 months prior to the study. These results align with studies conducted in Eastern Ethiopia and Ghana [13, 29] among female university students respectively.

Moreover, the current study found the following aspects to be motivators for family planning services utilisation: discussing FP with a sexual partner, persons an individual living with at home, adequate FP knowledge and positive perception towards FP use. In addition, the current study noted that the chance to utilise family planning services was two times higher among male participants compared to the female counterparts. As stated in the previous paragraph, the commonest method used was the male condom. This was thought to be influenced by many factors, such as its availability and accessibility.

Further, female participants may have been deterred to use FP by factors such as fear of side effects and sociocultural ties, as cited in the literature [26]. Similarly, this study’s result appear to be a new knowledge because majority of the reviewed studies did not indicate the association of male participants having the increased odds of contraceptive utilisation [15, 30–33].

The current study also has revealed that participants who were living with relatives other than their parents were two times more likely to use family planning services. This might had been happening because some parents have prohibitive social norms and traditional ties against FP use [28]. Existence of myths and misconceptions on contraceptive methods may be another reason for most of the parents to be a blocking stump against FP use among the youths. Some communities believe that contraceptive use promotes promiscuity and cheating [34, 35].

Parents with such conceptions may not be ready to let their sons and daughters use family planning methods. This underscores the need for comprehensive health education for the general community about the importance of family planning use and dispelling misconceptions. On the other hand, this study indicated that living with relatives other than parents significantly increased the odds of using family planning services. An in-depth exploratory study could be important in order to explore the factors, which made them to be more likely to be motivated and accept FP use among the youths than the reference category. Contrary to these current findings, a study from Kenya revealed that participants who were staying alone were more likely to utilise FP services than those who were staying with their parents [23].

Furthermore, the study shows that having adequate knowledge on contraceptive methods motivated the utilisation of the FP methods by almost two-folds compared to those who were not. This fact was supported by a study on married women on contraceptive utilisation, where the odds of utilisation increased by 16-folds [33, 36]. In addition, these findings are in line with [37, 38] who found that adequate FP knowledge increased the utilisation of the FP methods. However, contradictory findings showed that it did not translate into increased utilisation as reported by one study conducted among secondary school students in Dar es Salaam and Kilimanjaro [39, 40]. The study findings among female undergraduate university students in Kilimanjaro region in Tanzania confirmed that despite adequate knowledge on FP, the rate of FP use was low [15]. The conflicting knowledge about our findings and others might be contributed to by socio-demographic characteristic differences. Thus, a detailed and comprehensive study should be undertaken to evaluate why conflicting evidences exist towards one premise.

In addition, the current study has revealed that positive perception towards FP services increases the chance for utilisation by 1.772 times. These findings are in line with [41] who found that perceptions have significant effects towards FP use. Similar support was observed in the study conducted among married women in Mpwapwa Dodoma, where positive perceptions increased the odds of utilisation by over eight times [36].

Lastly, the findings also indicate that discussing FP matters with sexual partners increased the utilisation of FP services by more than six-times compared to those who did not. These findings are in line with [41, 42] who found that discussing FP with a sexual partner increased the utilisation. Similar findings were reported from a study conducted in Ethiopia [43]. With reference to previous literature, the knowledge of the partner on FP is very crucial for the discussion to be accepted by the other partner [38, 39, 42]. However, the male sexual partners tend to dominate the sexual relationship [44]. With reference to the findings of the current study, male partners’ FP knowledge was high. However, the current study’s findings are not in line with what was found by another study which reported that discussion with healthcare providers could be the only predictor for FP use among the youths [25, 35, 42]. This can be due to the type of predictors examined between this study and the reviewed study.

### Recommendations

There is urgent need of healthcare providers to conduct regular health promotion sessions on the family planning services to the youths at the clinic, learning institutions and to the community to raise awareness as well as to correct misconceptions and myths regarding FP services and promote their utilisation. In addition, increasing autonomy among female youth to be able to discuss family planning matters with their sexual partners is important. Families are encouraged to discuss the FP issues with their youth to enhance the uptake and avoid unwanted pregnancies and Sexually Transmitted Infections. This will make FP services to be understood by the students from their teen age for effective utilisation of the available FP methods. Furthermore, exploratory studies need to be conducted for in-depth understanding of the challenges facing the youths regarding FP utilisation.

## Conclusion

This study highlights a significant unmet need for family planning services among youth students in higher learning institutions in Dodoma, Tanzania, reflecting broader trends in Sub-Saharan Africa. Despite high sexual activity, family planning utilization remains suboptimal. Key motivators for using family planning services include living with relatives, having adequate knowledge, positive perceptions, and discussing family planning with sexual partners. These findings emphasize the need for targeted interventions focusing on education, fostering positive perceptions, and encouraging open partner discussions to enhance family planning utilization among adolescents, contributing to universal access to sexual and reproductive health services.

### Limitations

Based on this study, it is difficult to establish causal-effects relationship since the exposure and outcome measures are collected simultaneously. Also, the study has age limitation, since the participants’ age ranges were limited between 18–24 years, hence the results cannot be generalisable to students of other age groups. In addition, all information was based on the participants self-reports, which is prone to information bias. However, this potential was addressed by enlightening the participants about the purpose of the study and assuring them of confidentiality of the disclosed information.

## Supporting information

S1 Dataset (XLSX)
